# Metabolic Alterations in a Slow-Paced Model of Pancreatic Cancer-Induced Wasting

**DOI:** 10.1155/2018/6419805

**Published:** 2018-02-26

**Authors:** Elisabeth Wyart, Simone Reano, Myriam Y. Hsu, Dario Livio Longo, Mingchuan Li, Emilio Hirsch, Nicoletta Filigheddu, Alessandra Ghigo, Chiara Riganti, Paolo Ettore Porporato

**Affiliations:** ^1^Department of Molecular Biotechnology and Health Science, Molecular Biotechnology Center, University of Torino, Torino, Italy; ^2^Department of Translational Medicine, Istituto Interuniversitario di Miologia (IIM), University of Piemonte Orientale, Novara, Italy; ^3^Department of Molecular Biotechnology and Health Science, Molecular Imaging Center, University of Torino, Torino, Italy; ^4^Department of Oncology, University of Torino, Torino, Italy

## Abstract

Cancer cachexia is a devastating syndrome occurring in the majority of terminally ill cancer patients. Notably, skeletal muscle atrophy is a consistent feature affecting the quality of life and prognosis. To date, limited therapeutic options are available, and research in the field is hampered by the lack of satisfactory models to study the complexity of wasting in cachexia-inducing tumors, such as pancreatic cancer. Moreover, currently used *in vivo* models are characterized by an explosive cachexia with a lethal wasting within few days, while pancreatic cancer patients might experience alterations long before the onset of overt wasting. In this work, we established and characterized a slow-paced model of pancreatic cancer-induced muscle wasting that promotes efficient muscular wasting *in vitro* and *in vivo*. Treatment with conditioned media from pancreatic cancer cells led to the induction of atrophy *in vitro*, while tumor-bearing mice presented a clear reduction in muscle mass and functionality. Intriguingly, several metabolic alterations in tumor-bearing mice were identified, paving the way for therapeutic interventions with drugs targeting metabolism.

## 1. Introduction

More than half of cancer patients are suffering from a systemic wasting disorder referred to as cachexia (from Greek “bad condition”), a syndrome strongly affecting the quality of life and prognosis in cancer patients. This syndrome is characterized by unstoppable consumption of adipose and skeletal muscle tissues leading to an excessive body weight loss that cannot be fully reverted by conventional nutritional support [1].

Cancer cachexia is a complex syndrome accounting for multiple organ dysfunction and systemic metabolic deregulations [2]. Cachectic patients experience symptoms ranging from anorexia, elevated inflammation, and insulin resistance to increased energy expenditure, which ultimately promote malaise, fatigue, and impaired tolerance to chemotherapy [3], further worsening patients' prognosis. Besides being associated with a poor prognosis, cachexia is estimated to be the direct cause of one-third of cancer deaths [4]. Several tissue dysfunctions emerge during cachexia, such as liver steatosis, fat deposit lipolysis, intestinal dysbiosis, and, most notably, skeletal muscle wasting, which account for the steep decrease in quality of life, weakness, and respiratory distress of cancer patients.

Skeletal muscle atrophy is a highly regulated process driven by an unbalance between protein synthesis and degradation. Activation of the ubiquitin-dependent proteasome pathway (UPP) and the autophagy-lysosome system are two important mechanisms leading to increased protein breakdown. This process is orchestrated by a set of genes called *atrogenes*, such as Atrogin-1/MAFbx or Murf1 [5]. Compelling evidence shows that an impairment of mitochondrial metabolism and an increase in mitochondrial ROS are also strongly associated with the cachectic phenotype [6, 7].

Several tumor types, such as lung, gastrointestinal tract, and pancreatic cancer, are emerging as strong promoters of cancer cachexia [8]. In particular, pancreatic ductal adenocarcinoma (PDAC) presents a high penetrance of wasting, a process that seems to occur even in earlier phases of tumor transformation [9]. Despite the burden of cachexia in PDAC, there are still limited experimental models available.

Particularly, our understanding of the biology underlying cachexia is mostly based on the extensively used and well-characterized C26 carcinoma model, in which mice are drastically losing muscle and total body weight in a short period [10], thus contrasting with the progressive wasting occurring in the human pathology. It is known that the C26 model is associated with high levels of IL6 that play a central role in mediating muscle wasting [11], even though other inducers are probably involved in cachexia. In order to better characterize early stages of cachexia, we established a model of pancreatic cancer-induced cachexia able to promote mitochondrial metabolic alterations and a progressive wasting both *in vivo* and *in vitro*.

## 2. Materials and Methods

### 2.1. Animals

Young adult female C57BL/6J mice (9–12 weeks old) were used. All animal experiments were authorized by the Italian Ministry of Health and carried out according to the European Community guiding principles in the care and use of animals.

### 2.2. Generation of a PDAC Model

KPC tumor cells were derived from a primary culture of pancreatic tumor cells of the genetically engineered mouse model of PDAC (K-ras^LSL.G12D/+^; p53^R172H/+^; Pdx-Cre (KPC)).

0.7 × 10^6^ KPC cells in 200 *μ*l PBS were injected subcutaneously into the flank of C57bl/6J mice. Mice were sacrificed 5 weeks after injection, when tumor volume was approaching 5 mm of radius.

### 2.3. In Vivo Assessment of Muscular Strength

#### 2.3.1. Grip Test

An automatic grip strength meter was used to measure the maximum forelimb grip strength of mice. The machine measures the peak resistance force of the mouse while the latter is pulled away from the grid of the device. Each animal was assessed several times, and the final value corresponds to the average of 5 repeated force measurements in order to minimize procedure-related variability.

#### 2.3.2. Hanging Test

A wire-hanging test was used to assess whole-body muscle strength and endurance. The test was performed as previously described [12]. Briefly, mice were subjected to a 180-second hanging test on a wire, during which “falling” and “reaching” scores were recorded. When a mouse fell from the wire, the falling score was diminished by 1, and when a mouse reached one of the side of the wire, the reaching score was increased by 1. A final score was then established using both falling and reaching scores and was represented in the form of a Kaplan-Meier-like curve; scores have been normalized with respect to control.

### 2.4. MRI

Magnetic resonance images were acquired on a 1 Tesla M2 system (Aspect, Israel) equipped with a 30 mm transmitter/receiver (TX/RX) solenoid coil to determine body composition [13]. T_1_-weighted spin-echo images were acquired with high-resolution whole-body coronal orientation (repetition time/echo time/flip angle/number excitations [TR/TE/FA/NEX]: 400 ms/9.5 ms/90°/3, field of view [FOV]: 10 cm, matrix: 192 × 192, number of slices: 18, slice thickness: 1.5 mm, in-plane spatial resolution: 521 *μ*m, and acquisition time: 4 min). All T_1_-weighted images were processed by an in-house Matlab-developed script (MATLAB R2008, The MathWorks Inc.). The T_1_-weighted image histogram has three dominating classes, background, lean mass, and fat, so the total fat volume was isolated by segmenting the image into three categories by using a *k*-means clustering algorithm [14, 15].

### 2.5. Gene Expression Analysis

Gastrocnemii were harvested, frozen in liquid nitrogen, and crushed. Total RNA was extracted using TRIzol reagent (Invitrogen, Carlsbad, CA). cDNA was synthesized from 1000 ng of total RNA using cDNA reverse transcription kits (Applied Biosystems, Foster City, CA). Relative mRNA level was analyzed by real-time PCR (ABI 7900HT FAST Real-Time PCR system, Applied Biosystems, Foster City, CA) with TaqMan assays, using the Universal Probe Library system (Roche Applied Science, Penzberg, Germany). The 18S gene was used as a housekeeping control. The following primers were used: *FBX030 (MUSA1)*: F:5′-gagaagccagggtttgagc-3′ and R: 5′-tcatacagtgtgagtgctgctg-3′, *FBX032 (atrogin 1)*: F:5′-agtgaggaccggctactgtg-3′ and R: 5′-gatcaaacgcttgcgaatct-3′, *TRIM63 (MuRF1)*: F:5′-tgacatctacaagcaggagtgc-3′ and R: 5′-tcgtcttcgtgttccttgc-3′, and *cathepsin L* F:5′-caaataagaataaatattggcttgtca-3′ and R:5′-tttgatgtagccttccataccc-3′.

### 2.6. Western Blot

Protein samples from gastrocnemius were extracted with RIPA lysis buffer (150 mM NaCl, 50 mM Tris-HCl, 0.5% sodium deoxycholate, 1.0% Triton X-100, 0.1% SDS, and 1 mM EDTA) supplemented with protease and phosphatase inhibitor cocktail (Roche). Protein concentration was determined using the BCA protein assay (Thermo Fisher Scientific). Lysates were subjected to SDS-PAGE and then transferred to the PVDF membrane for immunoblotting analysis. The following antibodies were used: mono- and polyubiquitin (BML-PW8805, Enzo Life Sciences, 1 : 1000), p-AMPK (2535, Cell Signaling, 1 : 1000), and *β*-actin (4967, Cell Signaling, 1 : 1000).

### 2.7. Tissue Collection and Histology

Gastrocnemius muscle was excised, weighted, frozen in isopentane cooled in liquid nitrogen, and stored at −80°C. Transverse sections (7 *μ*m) from the medial belly were cut on a cryostat and collected on Superfrost plus glass slides. Cryosections were then processed for laminin staining. In detail, sections were fixed in 4% paraformaldehyde (PFA) for 10 min before being incubated with a laminin antibody (1 : 200; Dako) and visualized by an anti-mouse IgG Alexa Fluor 488 (Thermo Fisher Scientific) secondary antibody. Pictures of the whole slides were acquired with the slide scanner Pannoramic Midi 1.14 (3D Histech, Budapest, Hungary), and the cross-sectional area (CSA) was measured automatically by ImageJ software.

### 2.8. Succinate Dehydrogenase Activity

Succinate dehydrogenase (SDH) enzymatic activity was determined on 15 *μ*m cryosections by specific staining (Bio-Optica, Milan, Italy) following the producer's instructions. Briefly, the cryosections were incubated with the rehydrated SDH solution for 45 min at 37°C, washed, fixed, and mounted on slides. Images were then acquired with the slide scanner Pannoramic Midi 1.14.

### 2.9. Cell Culture and Conditioned Medium (CM) Preparation

C2C12 cells were cultured in DMEM/10% FBS and differentiated in DMEM/2% horse serum (HS) for 4 days as reported previously [16]. KPC cells were derived from a primary culture of pancreatic tumor cells of the genetically engineered mouse model of PDAC (K-ras^LSL.G12D/+^; p53^R172H/+^; PdxCre mice (KPC)).

Conditioned medium (CM) was prepared as follows: KPC cells were grown in DMEM with 10% FBS supplemented with 1% penicillin and streptomycin. When cells reached full confluence, the medium was removed; cells were washed twice with phosphate-buffered saline (PBS) and once with serum-free DMEM. Cells were grown in serum-free DMEM for further 24 h; then, the medium was collected, centrifuged at 4000 rpm for 10 min, aliquoted, and stored at −80°C. Atrophy on C2C12 was induced with 10% CM treatment for 48 h.

### 2.10. Myotube Diameter Quantification

C2C12 myotubes were treated with differentiation medium supplemented with 10% conditioned medium from KPC for 48 h. Pictures of myotubes were taken with bright field microscopy (Zeiss), and diameters of myotubes were measured using the software JMicroVision as previously described [16].

### 2.11. ROS Measurement *In Vitro*

ROS production was assessed in C2C12 myotubes by using the oxidant-sensitive fluorescent dye 29,79-dichlorodihydrofluorescein diacetate (H_2_DCFDA; Molecular Probes Inc., Eugene, OR). Cells were incubated with 10 *μ*M H_2_DCFDA in PBS for 30 minutes at 37°C under 5% CO_2_ atmosphere in darkness. An excess probe was washed out with PBS. Fluorescence was recorded at excitation and emission wavelengths of 485 nm and 530 nm, respectively, by a fluorescence plate reader (Promega). Fluorescence intensity was expressed as arbitrary units.

### 2.12. Mitochondrial Isolation

Mitochondrial fractions were isolated as previously reported [17], with minor modifications. Samples were lysed in 0.5 ml buffer A (50 mM Tris, 100 mM KCl, 5 mM MgCl_2_, 1.8 mM ATP, and 1 mM EDTA (pH 7.2)), supplemented with protease inhibitor cocktail III (Calbiochem), 1 mM PMSF, and 250 mM NaF. Samples were clarified by centrifuging at 650 ×g for 2 min at 4°C, and the supernatant was collected and centrifuged at 13,000 ×g for 5 min at 4°C. This supernatant was discarded, and the pellet containing mitochondria was washed in 0.5 ml buffer A and resuspended in 0.25 ml buffer B (250 mM sucrose, 15 mM K_2_HPO_4_, 2 mM MgCl_2_, 0.5 mM EDTA, and 5% BSA (*w*/*v*)). A 50 *μ*l aliquot was sonicated and used for the measurement of protein content or Western blotting; the remaining part was stored at −80°C.

### 2.13. Electron Transport Chain

The activity of complexes I–III was measured on 25 *μ*l of nonsonicated mitochondrial samples resuspended in 145 *μ*l buffer C (5 mM KH_2_PO_4_, 5 mM MgCl_2_, and 5% BSA (*w*/*v*)) and transferred into a 96-well plate. Then, 100 *μ*l buffer D (25% saponin (*w*/*v*), 50 mM KH_2_PO_4_, 5 mM MgCl_2_, 5% BSA (*w*/*v*), 0.12 mM cytochrome c-oxidized form, and 0.2 mM NaN_3_) was added for 5 min at room temperature. The reaction was started with 0.15 mM NADH and was followed for 5 min, and the absorbance was measured at 550 nm by a Synergy HT spectrophotometer (BioTek Instruments). Under these experimental conditions, the rate of cytochrome c reduction, expressed as nmol cyt c reduced/min/mg mitochondrial proteins, was dependent on the activity of both complex I and complex III [18].

### 2.14. Intramitochondrial ATP Levels

The amount of ATP was measured on 20 *μ*g of mitochondrial extracts with the ATPlite assay (PerkinElmer), according to the manufacturer's instructions. Data were converted into nmol/mg mitochondrial proteins, using a calibration curve previously set.

### 2.15. Intramitochondrial ROS Levels

The amount of ROS in mitochondrial extracts was measured fluorimetrically incubating mitochondrial suspension at 37°C for 10 minutes with 10 *μ*M of 5-(and-6)-chloromethyl-2,7-dichorodihydro-fluorescein diacetate-acetoxymethyl ester (DCFDA-AM) and then washed and resuspended in 0.5 ml of PBS. Results were expressed as nmol/mg mitochondrial proteins, using a calibration curve previously set with a serial dilution of H_2_O_2_.

### 2.16. Fatty Acid *β*-Oxidation

Long-chain fatty acids were measured as described by Gaster et al. [19] with minor modifications. 100 *μ*l mitochondrial suspension was rinsed with 100 *μ*l of 20 mM HEPES, containing 0.24 mM fatty acid-free BSA, 0.5 mM L-carnitine, and 2 *μ*Ci [1-^14^C]palmitic acid (3.3 mCi/mmol, PerkinElmer). Samples were incubated at 37°C for 1 h; then, 100 *μ*l of 1 : 1 phenylethylamine (100 mM)/methanol solution (*v*/*v*) was added. After one hour at room temperature, the reaction was stopped by adding 100 *μ*l of 0.8 N HClO_4_. Samples were centrifuged at 13,000 ×g for 10 min. Both the precipitates containing ^14^C acid-soluble metabolites (ASM) and the supernatants containing ^14^CO_2_ derived from oxidation (used as an internal control and expected to be less than 10% of ASM) were counted by liquid scintillation. Results are expressed as nmol/min/mg cellular proteins.

### 2.17. Statistics

Statistical significance was evaluated with one-way or two-way analysis of variance (ANOVA) for multiple groups, followed by a post hoc test as defined in the figure legends. Student's unpaired *t*-test was used to compare two groups. All error bars indicate SEM. Significance was established as *P* < 0.05. Data have been obtained from multiple independent experiments for an *in vitro* assay and from at least 4 mice for *in vivo* experiments. All the analyses were performed with the software PRISM5 (GraphPad Software).

## 3. Results

### 3.1. Establishment of a Slow-Paced Cancer-Induced Muscle Wasting Model

PDAC is known to induce muscle wasting with high penetrance [3]. Since cancer cachexia is a complex syndrome involving various pathological processes promoting wasting, such as anorexia and chronic inflammation, it is difficult to assess the direct contribution from the tumor. Therefore, we decided to assess the direct role of cancer cells in skeletal muscle atrophy via an *in vitro* model of atrophy, thus excluding other systemic confounding atrophic factors, hypothesizing that, in this type of cancer, atrophy can be mediated directly by tumor cell-secreted factors.

**Figure 1:**
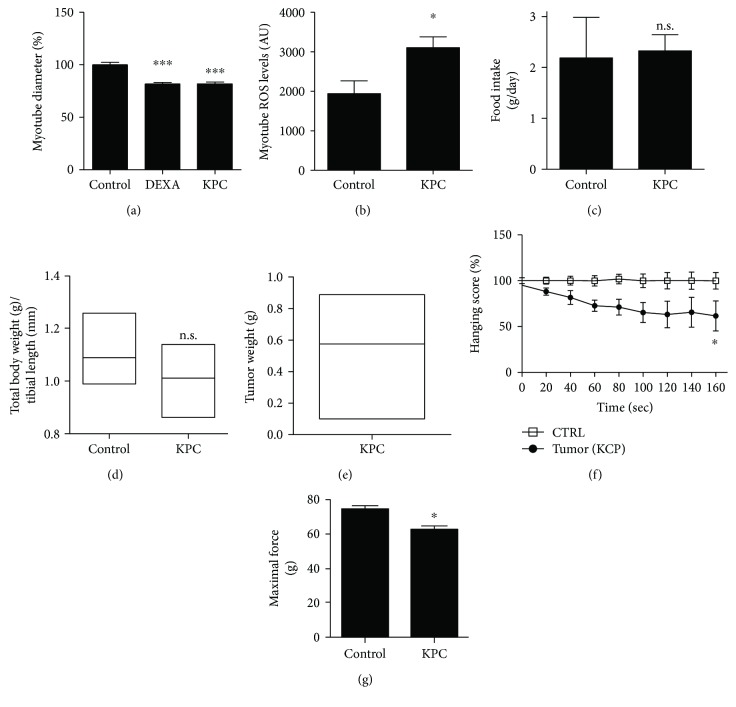
Characterization of an *in vitro* and *in vivo* model of cancer-associated atrophy. C2C12 myotubes treated for 48 h with 10% conditioned medium (CM) from KPC cells. Dexamethasone (DEXA) was used as a positive control of atrophy induction. Pictures were acquired at the brightfield microscope and (a) diameters were measured. (b) Increased ROS production in C2C12 myotubes treated with CM from KPC using oxidant-sensitive fluorescent dye H_2_DCFDA. (c) No difference in food intake in tumor-bearing versus control mice at the end of the experiment. (d) No significant weight loss in KPC tumor-bearing versus control mice. (e) Average tumor weight (min max box plot). Upon tumor growth at the end stage, mice presented reduced muscular resistance as evidenced by (f) performance at the hanging test (reach/fall assay) and (g) maximal strength as performed by the grip test of the upper limbs. All experiments have been performed with *N* ≥ 4 mice per group. All data are shown as means ± SEM; n.s. = nonsignificant; ^∗^*P* < 0.05; ^∗∗∗^*P* < 0.001; one-way ANOVA with Bonferroni correction for (a), two-way ANOVA with Dunnett's multiple comparison test for (e), and Student's *t*-test for (b), (c), (d), (f), and (g).

To this aim, we took advantage of KPC cells, a stable cell line derived from spontaneous primary tumor arising in C57BL/6 KRAS^G12D^ P53^R172H^Pdx-Cre^+/+^ (KPC) mouse [20], a genetically modified mouse model known to develop spontaneous wasting [9]. Similar to other cancer models [21], in our experimental conditions, KPC cells were able to directly promote muscle atrophy *in vitro*. Treatment of C2C12-derived myotubes with 10% KPC cell-conditioned media induced a consistent reduction in myotube thickness, similar to that elicited by dexamethasone, used as a positive control of atrophy induction (Figure 1(a)). A reduction in fiber thickness was associated with higher ROS generation (Figure 1(b)). A recent report from Michaelis et al. [20] showed that a subcutaneous injection of 5 million of these cells consistently promotes anorexia, hormonal dysfunctions, and lethal cachexia in 2 weeks. In order to establish a progressive model of wasting, we subcutaneously injected 0.7 million of KPC cells, which is the minimal amount able to consistently induce tumor growth without exacerbating factors such as excessive tumor burden and anorexia. Indeed, 5 weeks after KPC cell injection, neither food intake alteration (Figure 1(c)) nor macroscopic features of wasting were observed, despite a nonsignificant decreasing trend in body weight (Figure 1(d)). Tumor weight at the end of the experiment was approximatively 0.6 grams (Figure 1(e)), while in the other work, weight was between 1 and 2 grams [20].

Remarkably, despite the absence of overt signs of cachexia, skeletal muscle functionality, checked by rotarod evaluation twice a week (not shown), was drastically affected, but only at week 5, the week of the sacrifice. Accordingly, tumor-bearing mice showed reduced muscle performance, as assessed by the hanging-wire test [12] (Figure 1(f)), suggesting muscle deterioration in tumor-bearing animals. Along with reduced performance in stamina-related assays, mice displayed as well a reduction in grip strength, indicating also that the maximal force developed was reduced (Figure 1(g)).

**Figure 2:**
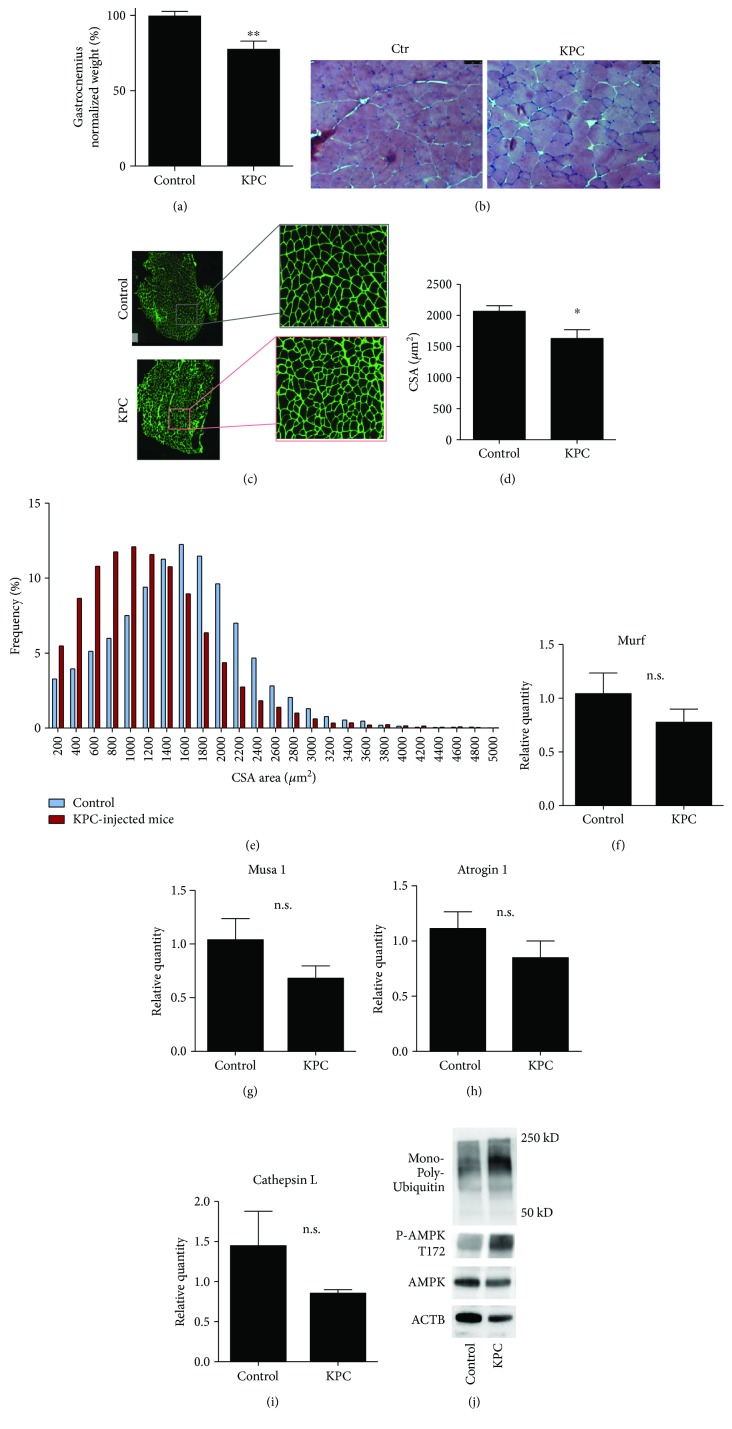
KPC cell injection promotes skeletal muscle atrophy. (a) Gastrocnemius weight normalized on tibial length. (b) H&E representative pictures of muscle sections from control and KPC-injected mice. (c–e) Gastrocnemius myofiber membranes were stained for laminin, pictures of the whole muscle section were acquired, and the cross-sectional area (CSA) was measured (c). (d) Frequency histogram showing distribution of myofiber CSA in control and KPC-bearing mice. *N* = 4. (e) Representative pictures of laminin staining for CSA analysis in control and KPC-bearing mice. (f–i) KPC injection does not upregulate atrogene expression. *N* ≥ 4. (j) Increased protein ubiquitination and AMPK (T172) phosphorylation in mice bearing tumor; blot representative of three independent experiments. All data are shown as means ± SEM. Statistical analyses were conducted using two-tailed *t*-test. n.s. = nonsignificant.

Coherently with the decrease in muscle functionality, 5 weeks after KPC injection, mice presented a consistent loss of gastrocnemius mass of roughly 20% (Figure 2(a)). The decrease was related to a reduction in average fiber size as detailed by histological analysis (Figures 2(b)–2(d)). Coherently, analysis of fiber cross-sectional area (CSA) distribution highlighted a shift towards smaller areas (Figure 2(e)). A reduction in muscle mass was not associated with transcriptional regulation of atrogenes Atrogin1, Musa, and Murf1 and of cathepsin L (Figures 2(f)–2(i)), nor with altered expression of ATG7, BECLIN1, and LC3 in gastrocnemii (not shown). Nevertheless, muscle protein lysates presented increased protein ubiquitination (Figure 2(j)), indicative of an activation of the UPP. Along with increased protein ubiquitination, we identified higher AMPK phosphorylation, in line with the emerging role of AMPK as a functional player in cancer cachexia [22].

### 3.2. Mice Undergoing Muscle Dysfunction Present Altered Lipid Metabolism

**Figure 3:**
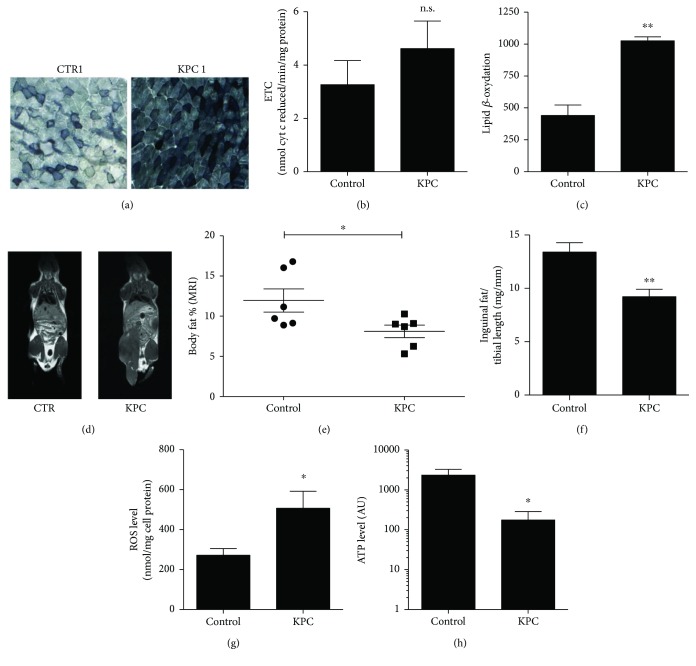
Metabolic dysregulation of skeletal muscle in KPC-bearing mice. (a) Representative images for succinate dehydrogenase (SDH) activity stain. (b) Gastrocnemius mitochondria were isolated, and ETC activity from complex I to complex III was assessed by evaluating cytochrome c reduction. (c) ^14^C-labeled palmitate was used as a substrate to measure lipid beta-oxidation in isolated mitochondria of gastrocnemius from control and KPC-bearing mice. (d) Representative T_1_-weighted MR images (brightest regions in T_1_-weighted MR images correspond to adipose regions) and (e) in vivo measurement of adipose tissue using MR images in the control group versus KPC-bearing mice. (f) Weight of inguinal fat normalized on tibial length for control versus KPC-bearing mice. (g) Gastrocnemius mitochondria were isolated, and ATP level was assessed using the ATPlite kit (PerkinElmer, USA). (h) ROS measurement in isolated mitochondria from gastrocnemius using H_2_DCFDA. N ≥ 4. All data are shown as means ± SEM. Statistical analysis was conducted using two-tailed *t*-test. n.s. = nonsignificant; ^∗^*P* < 0.05; ^∗∗^*P* < 0.01.

Given the importance of energy metabolism in regulating skeletal muscle mass and functionality [23, 24], we investigated potential alterations of mitochondrial metabolism in the skeletal muscle of KPC-bearing mice. To this aim, we assessed basal complex II activity in gastrocnemii by performing the succinate dehydrogenase (SDH) activity assay. Intriguingly, gastrocnemius sections from KPC-bearing animals presented increased complex II activity, as evidenced by the increased concentration of blue tetrazolium salt (Figure 3(a)). However, this increased activity was not coupled with elevated flux through the electron transport chain (ETC). Indeed, ETC, as measured by cytochrome c reduction rate in uncoupled mitochondria, was similar in the two groups (Figure 3(b)).

While SDH activity supports ETC, it is also part of the tricarboxylic acid (TCA) cycle and it is linked to fatty acid oxidation, allowing ketone bodies generated by acetyl coenzyme A due to excessive fatty acid oxidation to enter the TCA.

To clarify whether the increased SDH activity was indicative of increased fatty acid oxidation, we measured this metabolic pathway in isolated mitochondria from the gastrocnemius of either control or KPC-bearing mice and we observed a significant increase in fatty acid oxidation in muscles of tumor-bearing mice (Figure 3(c)), consistent with the increase in complex II activity.

In order to identify if the altered intramuscular lipid oxidation was correlated with a systemic dysregulation during this precachectic process, we performed T_1_-weighted magnetic resonance imaging (MRI). Intriguingly, 4 weeks post-KPC injection (one week before sacrifice), precachectic mice presented reduced bright hyperintensity regions, indicative of reduced fat deposits (Figures 3(d) and 3(e)).

Coherently, at the time of sacrifice, KPC-bearing mice presented a significant reduction in inguinal fat tissue mass (Figure 3(f)). Therefore, we speculated that the reduced fat content might be related to increased fatty acid oxidation, a feature previously associated with cancer cachexia [23] in other tumor types.

High SDH activity [25, 26] and excessive fatty acid oxidation might lead to ROS accumulation [27], ultimately promoting mitochondrial dysfunction [28] and fiber damage. Hence, we investigated the impact of tumor growth on mitochondrial ROS and energetic balance. Mitochondria extracted from KPC-bearing animals had indeed increased ROS (Figure 3(g)), coupled with reduced ATP (Figure 3(h)), suggesting that the increased fatty acid oxidation may have a detrimental rather than beneficial effect on mitochondria.

## 4. Discussion

Pancreatic cancer is a pathology with dismal prognosis associated with a stark decrease in quality of life, mostly because of cachexia development [29, 30]. While cachexia is considered the last step of cancer progression, it is important in PDAC to model the earliest steps of the disease (i.e., precachexia). Indeed, Mayers et al. [9] found that spontaneous PDAC mouse model presents an increased release of amino acids from the skeletal muscle months before the development of cachexia, which is consistent with the data from PDAC patients [9]. These data advocate for the importance of defining alterations in skeletal muscle occurring in the early phases of disease, before the establishment of overt cachexia.

To this aim, we modified the cancer cachexia model described by Michaelis et al. to reproducibly induce cachexia with KPC cells [20]. Since KPC-bearing male mice present hormonal dysfunctions, we performed the study in female mice, although these animals are characterized by a moderate degree of wasting. While Michaelis and coworkers modeled cachexia by injecting up to 5 × 10^6^ cells per mouse, thus promoting anorexia and subsequent animal death starting from 11–14 days, we injected only 0.7 × 10^6^ cells (the minimal amount necessary to consistently promote tumor growth) in order to promote a slower tumor growth, thus allowing the characterization of precachectic events. This reduced cell number resulted in barely palpable tumors at 2 weeks after injection (the time point where mice from Michaelis et al. already started to die of cachexia). Moreover, in contrast to the effects reported with the injection of higher amount of KPC cells, smaller tumor mass (up to 75% reduction) and no effect on heart weight were observed (data not shown).

Despite the moderate degree of wasting, mice presented reduced muscle function and strength. In order to detect alterations in muscle morphology occurring at early phases of wasting, we sacrificed the mice at the first sign of decreased performance. At necropsy, mice presented a statistically significant reduction in gastrocnemius weight and fat deposit, but no signs of anorexia, excluding the involvement of food intake in the skeletal muscle mass reduction. Intriguingly, we found only a trend towards decreased body weight, similar to the findings of Brown and colleagues [7]. Of note, it is known that many tumors hijack organ function, especially the liver and spleen [3], by inducing increased organ size, which might counterbalance the drop in muscle and fat in the first phases. Muscle from tumor-bearing mice did not present any major transcriptional regulation of the investigated atrogenes. However, we identified increased levels of AMPK^T172^ phosphorylation, indicative of ongoing energy stress, and protein ubiquitination indicating that the alterations present in muscle were mediated by a different pathway, that is, alternative ubiquitin ligase, a regulation at protein level, or alterations in protein deubiquitinases.

As previously shown in clear cell kidney cancer, muscle undergoing wasting is causally linked to increased fatty acid oxidation [23, 31], potentially raising noxious ROS generation in the mitochondria. Noteworthy, also in our model, the significant drop in lipid was coupled with an increased fatty acid utilization and mitochondrial ROS generation, indicating a potential source of oxidative stress causing reduced muscular function and degeneration. Intriguingly, we identified an increased activity of SDH, uncoupled with increased ETC flux. Moreover, ATP content was decreased, suggesting a profound mitochondrial alteration. This observation further supports the concept that mitochondrial alterations occur at the early phases of cachexia. While SDH does not contribute to increasing mitochondrial energy metabolism in cachectic muscles, it promotes the metabolism of ketone body derivatives that are produced in conditions of high fatty acid oxidation, that is, in the same metabolic conditions of KPC-bearing muscles. Redox cycles occurring at complex I and complex III of the ETC are generally considered the key sources of ROS within mitochondria. However, also, the SDH complex has been recognized as an important source of intramitochondrial ROS [25]. Taken together, our findings suggest that muscles consume fatty acids, forcing SDH activity in early cachexia. The final result is an energetic catastrophe that may severely impair muscle physiological performance.

Interestingly, *in vitro* myotubes did not show increased fatty acid oxidation during atrophy (not shown), in line with the fact that culture and differentiation media contain limited amount of fatty acids. However, C2C12 cells treated with the medium of pancreatic cancer cells displayed the same alterations observed in cachectic muscles, notably high ROS levels and AMPK phosphorylation (not shown), suggesting that common metabolic alterations in mitochondrial metabolism occur in the early phase of cachexia both *in vitro* and *in vivo*.

In conclusion, we report a novel model of precachexia causing a drastic reduction in muscle function and an initial reduction in skeletal muscle mass. Interestingly, the onset of increased fatty acid oxidation and mitochondrial ROS generation occurs before the emergence of muscle mass reduction. Further test inhibiting fatty acid oxidation or mitochondrial ROS generation will be instrumental in understanding the relative contribution of such pathways to the pathogenesis of cachexia, as well as in the identification of the factors secreted by PDAC cells causing muscle atrophy, both *in vitro* and *in vivo*.
